# Vitamin D may alleviate pre‐eclampsia by modulating the ferroptosis signalling pathway: A hypothesis based on recent literature

**DOI:** 10.1111/jcmm.17754

**Published:** 2023-04-26

**Authors:** Cui‐Ping Li, Hua‐Qin Su, Lian‐Ping He

**Affiliations:** ^1^ School of Medicine Taizhou University Jiaojiang Zhejiang China; ^2^ Taizhou Jiaojiang District Xiachen Street Community Health Service Centre Jiaojiang Zhejiang China

**Keywords:** ferroptosis, pre‐eclampsia, signalling pathways

## Abstract

Ferroptosis is a novel form of regulated cell death typically characterized by non‐apoptotic, iron‐dependent, and reactive accumulation of oxygen species. Recent studies have found that ferroptosis plays an important role in the pathophysiology of pre‐eclampsia (PE). In order to find potential therapeutic targets for ferroptosis intervention and better prevent the occurrence and progression of PE, the signalling pathways that regulate ferroptosis need to be identified. In this article, we review the role of vitamin D in PE and the role of ferroptosis in PE. Based on recent literature, we propose the scientific hypothesis that vitamin D can alleviate preeclampsia by modulating the ferroptosis signalling pathway. The aim of this review is to understand the regulatory pathways of ferroptosis in PE and to identify potential therapeutic targets.

## 1 INTRODUCTION

**FIGURE 1:**
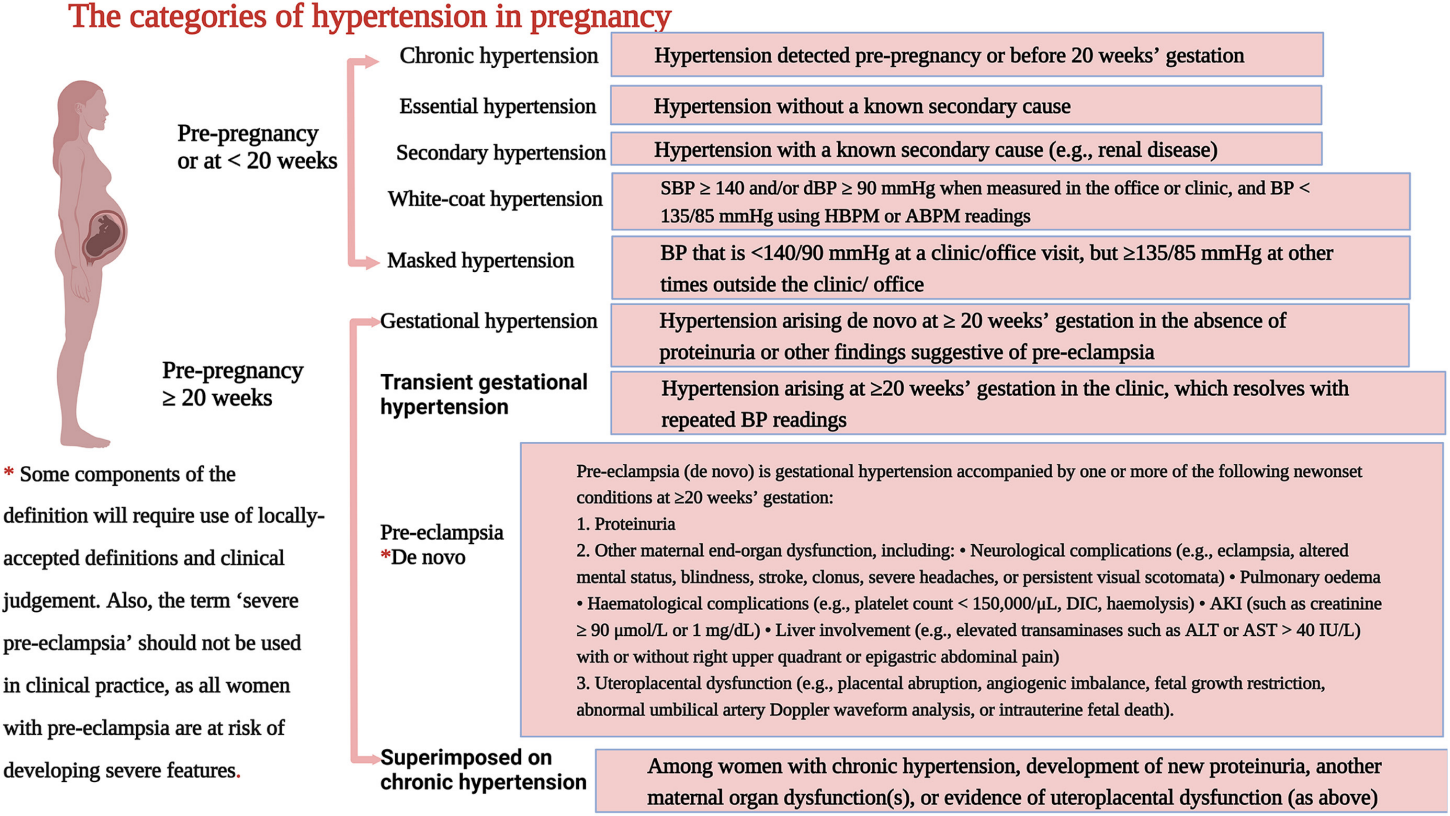
Classification of hypertension in pregnancy.

Pre‐eclampsia (PE) is a major complication of pregnancy and is a significant burden to maternal and foetal health. A registry‐based nationwide controlled cohort study found that PE significant elevated cardiovascular disease (CVD) risks of Finnish women.^1^ A meta‐analysis involving 39 million women from 40 countries showed that the overall estimates are 4.6% of all deliveries for preeclampsia.^2^ PE is difficult to define because it is a syndrome characterized by many clinical manifestations. Their co‐existence leads to diagnosis and treatment. There is no gold standard, all functions are non‐specific. Numerical features such as blood pressure and proteinuria are defined by arbitrary thresholds. Thus, although these definitions appear to be accurate, they are unwarranted and there is still a great deal of uncertainty. Until recently, the accepted definition of PE was new‐onset hypertension and proteinuria that developed in the second half of pregnancy and resolved after delivery. A more common and less dangerous form of new‐onset hypertension without proteinuria is called gestational hypertension.^3^ Figure 1 shows the international society for the study of hypertension in pregnancy (ISSHP)‐endorsed classification of hypertension in pregnancy.^4^

Current management of PE includes pre‐counselling, prenatal blood pressure screening, patient‐performed home blood pressure monitoring,^5^ peripartum blood pressure control, management of complications, timely delivery, and monitoring.^6^ Various approaches have been used to reduce the risk of PE in susceptible women, such as calcium supplementation, antioxidant therapy, and antiplatelet therapy,^7^ but the only proven treatment for BE is childbirth. Close observation of PE patients without severe symptoms before 37 weeks of gestation is recommended, and delivery is recommended at 37 weeks of gestation.

## 2 FERROPTOSIS AND PE

Ferroptosis, a new form of regulated cell death, is usually characterized by non‐apoptotic, iron‐dependent, and the accumulation of reactive oxygen species (ROS).^8^ Ferroptosis with unique morphological and bioenergetic features is also a distinct form of cell death, which is different from other already established cell death. Morphological features of ferroptosis mainly occur in mitochondria, such as reduced mitochondria, increased mitochondrial membrane density, damaged or absent mitochondrial cristae, and rupture of the outer mitochondrial membrane, but the nuclear morphology remains normal. Bioenergetic properties of ferroptosis are mainly iron accumulation and lipid peroxidation. So far, ferroptosis has been found in many diseases and plays an important role in the occurrence and development of many diseases. Related diseases modifiable by ferroptosis was reviewed in details.^9^

**FIGURE 2:**
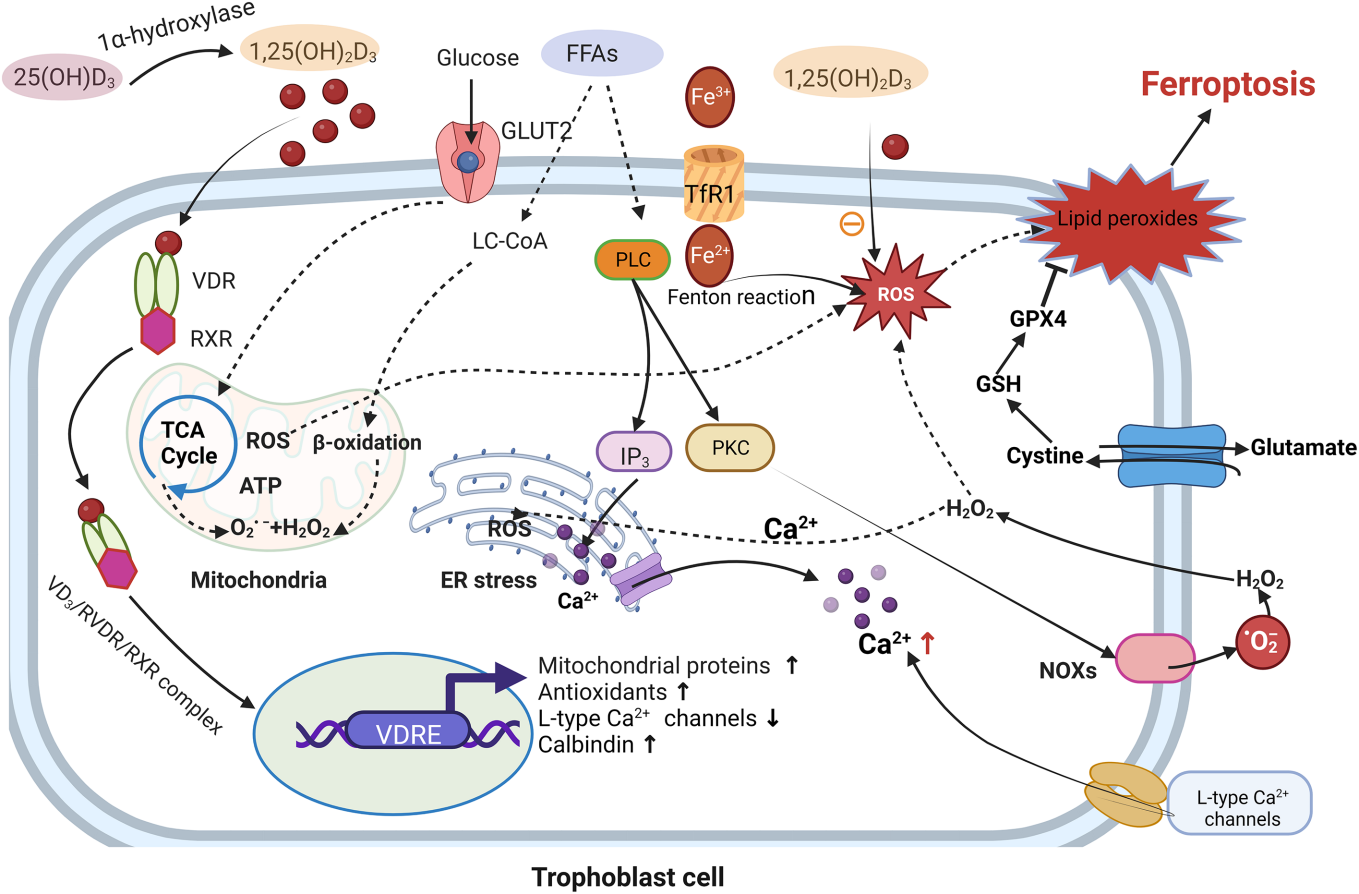
Signalling pathways implicated in ferroptosis.

In particular, ferroptosis is involved in the pathological process of PE. Study found that ferroptosis in trophoblasts via the ferritinophagy pathway implicated in the pathogenesis of PE.^10^ Reviews of the relationship between PE and ferroptosis have been reported.^11^, ^12^ In this article, we systematically summarize the pathways of ferroptosis and elaborate on the role of ferroptosis in PE, so as to provide intervention strategies for the pathways of ferroptosis in PE (Figure 2).

## 3 VITAMIN D AND PE

Accumulating epidemiological studies support that Vitamin D deficiency is associated with the pathogenesis of preeclampsia.^13^ Meanwhile, vitamin D deficiency is also associated with type 1 diabetes,^14^ obesity,^15^ and irritable bowel syndrome.^16^ There is extensive literature on vitamin D deficiency increasing the risk of preeclampsia.^17^ In a case–control study, disrupted vitamin D homeostasis and calcium transport was found in the placenta of PE cases.^18^ A systematic review and meta‐analysis found that micronutrient supplementation (calcium and vitamin D) interventions reduced the risk of developing PE.^19^ Vitamin D is known to promote calcium absorption. Can calcium supplementation also reduce the risk of PE? A systematic review and network meta‐analysis revealed that low‐ and high‐dose calcium supplementation are effective for PE prevention in women with low calcium intake.^20^

But there are also literature reports that vitamin D receptor gene polymorphism and vitamin D deficiency are not associated with the risk of preeclampsia.^21^ This study was inconsistent with other studies, possibly due to the small sample size. In that case–control study, 40 preeclamptic and 40 normotensive pregnant women were compared for vitamin D status and vitamin D receptor gene polymorphism.^21^ Taken together, there is more evidence that vitamin D or calcium supplementation can reduce the risk of PE.

## 4 VITAMIN D AND FERROPTOSIS

There is no direct evidence that vitamin D supplementation reduces the risk of PE by modulating ferroptosis. Previous reviews have provided comprehensive coverage of the role of vitamin D and calcium supplementation in ferroptosis.^22^ Calcium metabolism is closely related to iron metabolism. A review details that calcium‐iron connection plays an important role in eisenfohr‐mediated neuronal cell death.^23^ Apparently, calcium inhibits the absorption of iron in the gut. Calcium is a noncompetitive inhibitor of ferrous transporter 1 (DMT1) during intestinal iron absorption.^24^ Calcium also induces the downregulation of genes related to iron absorption.^25^ Another study also found that a low‐calcium diet increased tissue iron levels in ovariectomized rats.^26^ A case–control study found that total and ionized calcium were inversely associated with iron overload in children with transfusion‐dependent thalassemia.^27^ It is well known that iron overload is an important marker of ferroptosis. In conclusion, an imbalance in calcium homeostasis may be one of the most important causes of ferroptosis. Vitamin D supplementation can promote calcium absorption. One study suggested that oxidative stress may be associated with disruption of vitamin D homeostasis and placental calcium transport in PE patients.^28^ Intake of calcium and potassium may play an important role in PE.^29^ It is reasonable to speculate that vitamin D supplementation may inhibit iron absorption by promoting calcium absorption, and ultimately inhibit the occurrence of ferroptosis. Based on recent literature, we propose the scientific hypothesis that vitamin D can alleviate preeclampsia by modulating the ferroptosis signalling pathway.

## 5 CONCLUSIONS AND FUTURE PERSPECTIVES

Ferroptosis is a newly defined type of RCD driven by the accumulation of iron‐dependent lipid ROS and associated with the development of many diseases. In pregnant women, ferroptosis affects placental function, participates in the occurrence of PE and other unexpected pregnancy diseases, and seriously threatens the health of mothers and babies. Common mechanisms of ferroptosis in PE include iron overload, lipid peroxide hyperplasia, GPX4 inhibition, and systemic Xc‐inhibition. Calcium is a noncompetitive inhibitor of ferrous transporter 1 (DMT1) during intestinal iron absorption. Calcium also induces the downregulation of genes related to iron absorption. It is speculated that vitamin D supplementation may inhibit iron absorption by promoting calcium absorption, and ultimately inhibit the occurrence of ferroptosis. There are few studies on the role of iron‐calcium connection in PE. Therefore, whether iron‐calcium connection play an important role in iron–iron‐induced PE needs further investigation.

At present, there are many studies on the treatment of PE, and many drugs based on placental oxidative stress have been proven effective, such as aspirin. However, there is currently no definitive treatment for ferroptosis in PE. Therefore, more siderochondria‐specific treatments are urgently needed for the benefit of patients. Likewise, research on biomarkers of ferroptosis in PE is needed, as it facilitates early detection and diagnosis of the disease and predicts the severity of the disease. In addition, the role of the iron‐calcium connection signalling pathway in ferroptosis remains to be further studied. Therefore, the role of the iron‐calcium complex signalling pathway as a new therapeutic target in ferroptosis‐induced PE needs further investigated.

## AUTHOR CONTRIBUTIONS

**Cui‐Ping Li:** Writing – original draft (equal). **Hua‐Qin Su:** Writing – review and editing (equal). **Lian‐Ping He:** Writing – review and editing (equal).

## FUNDING INFORMATION

This work was supported by the 2022 Taizhou University Higher Education Teaching Reform Project (No. 105 and No. 114).

## CONFLICT OF INTEREST STATEMENT

The authors declare that they have no conflicts of interest.

## Data Availability

The datasets generated and analysed during the current study are available from the corresponding author upon reasonable request.
